# Dexamethasone-loaded platelet-inspired nanoparticles improve intracortical microelectrode recording performance

**DOI:** 10.21203/rs.3.rs-6018202/v1

**Published:** 2025-02-14

**Authors:** Andrew Shoffstall, Longshun Li, Aniya Hartzler, Dhariyat Menendez-Lustri, Jichu Zhang, Alex Chen, Danny Lam, Baylee Traylor, Emma Quill, George Hoeferlin, Christa Pawlowski, Michael Bruckman, Sen A. Gupta, Jeffrey Capadona

**Affiliations:** Case Western Reserve University; Case Western Reserve University; Case Western Reserve University; Case Western Reserve University; Case Western Reserve University; Case Western Reserve University; Case Western Reserve University; Haima Therapeutics; Haima Therapeutics; Case Western Reserve Unviersity; Haima Therapeutics; Haima Therapeutics; Case Western Reserve; Case Western Reserve University

**Keywords:** Intracortical microelectrodes, Neuroinflammation, Dexamethasone sodium phosphate, Lipid-based nanoparticles, Targeted drug delivery, Brain machine interfacing, Neural engineering, Artificial platelets

## Abstract

Long-term robust intracortical microelectrode (IME) neural recording quality is negatively affected by the neuroinflammatory response following microelectrode insertion. This adversely impacts brain-machine interface (BMI) performance for patients with neurological disorders or amputations. Recent studies suggest that the leakage of blood-brain barrier (BBB) and microhemorrhage caused by the IME insertions lead to the increased neuroinflammation and reduced neural recording performance. Additionally, a sustained presence of activated platelets and coagulation factors is found near the insertion site. Thus, we hypothesized that the systemic administration of dexamethasone sodium phosphate-loaded platelet-inspired nanoparticle (SPPINDEX) can improve the neural recording performance of intracortical microelectrodes (IMEs) by promoting hemostasis, facilitating blood-brain barrier (BBB) healing, and achieving implant-targeted drug delivery. Leveraging the hemostatic and coagulation factor-binding properties of the platelet-inspired nanoparticle (PIN) drug delivery platform, SPPINDEX treatment can initially attenuate the invasion of neuroinflammatory triggers into the brain parenchyma caused by insertion-induced microhemorrhages or a compromised BBB. Furthermore, targeted delivery of the anti-inflammatory drug dexamethasone sodium phosphate (DEXSP) to the implant site via these nanoparticles can attenuate ongoing neuroinflammation, enhancing overall therapeutic efficacy. Weekly treatment with SPPINDEX for 8 weeks significantly improved the recording capabilities of IMEs compared to platelet-inspired nanoparticles alone (PIN), free dexamethasone sodium phosphate (Free DEXSP), and a diluent control trehalose buffer (TH), as assessed through extracellular single-unit recordings. Immunohistochemical analyses of neuron density, activated microglia/macrophage density, astrocyte density, and BBB permeability suggest that the improved neural recording performance may be attributed to reduced neuron degeneration, activated microglia and astrocytes at the implant interface caused by the decreased infiltration of blood-derived proteins that trigger neuroinflammation and the therapeutic effects from DEXSP. Overall, SPPINDEX treatment promotes an anti-inflammatory environment that improves neuronal density and enhances recording performance.

## Introduction

Intracortical microelectrodes (IMEs) are devices designed to record neuronal activity for neuroscience research and the treatment of neurological disorders [1–4]. Neural signals acquired by IMEs can be analyzed and decoded for brain-machine interface (BMI) applications, such as controlling external devices, prosthetics, and stimulators to restore motor function in patients with limb loss or spinal cord injuries [5–10]. However, the primary challenge for the clinical translation of BMI technology is the lack of long-term reliability and stability of IMEs [11–17]. The brain’s neuroinflammatory response following IME insertion is widely recognized as a major cause of the decline in neural recording performance over time [4, 18, 19].

Upon the insertion of IMEs into brain motor cortex, healthy neurons and the blood-brain barrier (BBB) are disrupted, leading to an initial reduction in neural recording cellular resources and the influx of blood-derived factors into the brain parenchyma [13, 15, 18, 20–27]. The disruption induces neuron death, and the infiltration of blood proteins triggers the first activation and recruitment of resident microglia, which produce proinflammatory cytokines and cytotoxic factors. This, in turn, leads to the recruitment of astrocytes and secondary neuron death[28–31]. The secondary neuron death further perpetuates neuroinflammation, resulting in the encapsulation of microglia and astrocytes around IME interface, which diminishes the neural recording performance of IMEs [32–36]. Additionally, activated platelets and coagulation factors such as von Willebrand Factor (vWF), collagen, fibrinogen have been observed to be exposed and/or infiltrated near IMEs interface, potentially contributing to neuroinflammation up to at least 8 weeks [37].

Other groups in the field have attempted to reduce the sequelae of neuroinflammation using anti-inflammatory or anti-oxidative therapies after IME implantation. The approaches have ranged from systemic administration, local administration, and device modifications, including coatings, retrodialysis and others [16, 17, 28, 29, 38–44]. For example, Gaire et al have demonstrated that systemic administration of dexamethasone (DEX) acutely post IMEs insertion may not improve the recording functionality of IMEs, even though it attenuated neuroinflammation [38]. Furthermore, high-dose systemic administration of DEX is contraindicated due to potential adverse effects, including reduced bone mineral density, altered glucose levels, and impaired organ function [45]. Zhong *et al.* have previously encapsulated dexamethasone into a nitrocellulose coating on the microelectrodes to achieve local release of drugs around the implantation site, which resulted in significant reduction of neuroinflammation and improvement in signal recording quality [39]. However, local delivery with DEX coated IMEs is not ideal and limited to single application of medicine after surgery since DEX coating will be entirely consumed in days. Moreover, direct modification of the IMEs could result in additional commercial and regulatory burden for integration with existing devices. Although extensive studies have focused on addressing IME insertion-induced neuroinflammation to improve recording stability, few proposed therapies specifically targeting the initial prevention of foreign substance invasion into the brain parenchyma or achieving repeatable and targeted delivery of anti-inflammatory agents to the IME implant site.

Platelet-inspired nanoparticles (PIN) have demonstrated the ability to attenuate the invasion of blood-derived proteins and achieve localized delivery near the IME implant site by colocalizing with activated platelets and enhancing hemostasis in response to IMEs insertion-induced microhemorrhage [46]. At a vascular injury site, PINs can bind to exposed collagen and vWF via their collagen- and vWF-binding peptides (CBP and VBP), and to activated platelet GPIIb-IIIa receptors via fibrinogen-mimetic peptides (FMP), thus actively accumulating at the injury site [47–49]. Therefore, we hypothesize that PIN can be used to targeted deliver anti-inflammatory drug dexamethasone sodium phosphate (DEXSP) to the IME implant site, thereby attenuating insertion-induced neuroinflammation. In combination with its function to promote hemostasis and reseal compromised BBB, reducing sources that trigger neuroinflammation, systemic administration of the multifunctional DEXSP-loaded PIN (SPPINDEX) therapy is expected to significantly reduce neuroinflammation and enhance the neural recording performance of IMEs.

Our study aimed to evaluate the therapeutic efficacy of systemically administered SPPINDEX on the long-term IME electrophysiology recording quality. First, we investigated the feasibility of manufacturing SPPINDEX with high encapsulation efficiency to achieve an effective dosage of DEXSP under a limited particle dosage, using lipid thin-film rehydration with DEXSP-containing solution. An *in vitro* release study was conducted to characterize the drug release profile from PIN. We then assessed the IME recording performance of neuronal activity under weekly treatment over an 8-week period using extracellular single-unit recording. To further understand the therapeutic effect of SPPINDEX on neural recording performance, we quantified neuron density (NeuN) near the implantation site at the 8-week endpoint to verify neural health and viability. Additionally, activated microglia (CD68) and astrocytes (GFAP) were evaluated to assess neuroinflammation. The ability of SPPINDEX to reduce blood-derived protein invasion was characterized by immunoglobulin G (IgG) staining. Finally, to ensure that repeated weekly dosing of SPPINDEX over 8 weeks did not induce adverse effects on metabolic homeostasis, kidney and liver functions, or complications such as femur osteoporosis, we monitored rodent weight, glucose (GLU), alanine transaminase (ALT), creatinine (CREA) levels, and femur bone mineral density (BMD).

## Materials and Methods

### Intracortical Microelectrode Validation and Sterilization

The quality of 16-channel, single-shank intracortical microelectrode (A1×16–3mm-100–177-Z16, NeuroNexus, Ann Arbor, MI) was validated using electrochemical impedance spectroscopy (EIS) assays. A Gamry Interface 1010E Potentiostat was employed to perform EIS on each electrode site of the IME, using the electrode site as the working electrode, Ag | AgCl electrode as the reference and a platinum wire as the counter electrode. EIS measurements were conducted in 1 × PBS electrolyte (pH 7.5) and all wire connections placed inside a Faraday cage. Impedances at each electrode site were tested across a frequency range of 1 HZ to 10^6^ Hz (12 points per decade) in a 50 mV rms AC voltage. The 1 kHz impedance was specifically measured to validate the quality of IME prior to *in vivo* single-unit electrophysiology recording. IMEs with normal 1 kHz impedance values (between 100 kΩ and 1 MΩ) across all 16 electrode sites were deemed suitable for IME implantation surgery. Validated IMEs and 2 mm × 123 μm × 15 μm non-functional Michigan-style single-shank silicon microelectrodes (Qualia Labs, Dallas, TX) were sterilized by Ethylene Oxide (EtO) prior to surgeries.

### Intracortical Microelectrode Implantation Surgery

All studies described herein were reviewed and approved by the Institutional Animal Care and Use Committee (IACUC) at the Louis Stokes Cleveland Department of Veteran Affairs Medical Center, and were performed in accordance with the ARRIVE guidelines. 29 male rats with indwelling vascular catheters (Sprague Dawley, CD, Charles River Labs, Wilmington, MA) weighing 250–280 grams were included in this study. Surgeries to implant vascular catheters were performed by Charles River Labs. In this study, polyurethane catheters were inserted into the carotid artery and advanced the aortic arch for weekly dosing of treatments which was described in our previous study [46]. All rats were housed up to 1 week prior to further procedures.

Surgical procedures for implanting functional intracortical microelectrodes (IMEs) in the rat cortex were adapted from previously published methods [22, 46, 50, 51]. All rats were initially anesthetized with 3.5% isoflurane and maintained at a surgical level of anesthesia with 1.5–3% isoflurane throughout the procedure. After ensuring anesthesia, eye ointment was applied to protect the corneas, and the nails were clipped to reduce the risk of premature removal of postoperative sutures. The surgical site was shaved, and the animals received subcutaneous injections of cefazolin (16 mg/kg) and meloxicam (1 mg/kg) to prevent infection and manage pain, respectively. Additionally, 0.2 mL of 0.25% Marcaine was administered locally to the incision site for localized pain relief. Following these preparations, the rat was secured in a stereotaxic frame using blunt ear bars and an incisor bar. Vital parameters, including SpO2, heart rate, and body temperature, were continuously monitored throughout the surgery. Regular toe pinches and observations of paw color were performed to confirm the maintenance of an adequate anesthetic plane. The incision area was sterilized with alternating applications of Betadine and isopropyl alcohol.

**Figure 1:**
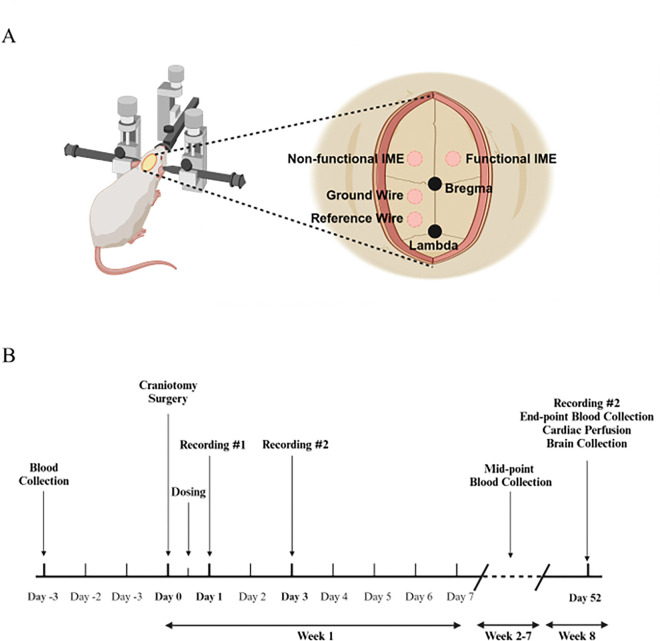
Schematic of craniotomy, implantation, and study timeline. (A) Craniotomies are performed at the labeled sites, showing different types of implantations: non-functional IME, functional IME and bone screws for ground and reference wires. Non-functional IMEs were inserted for increasing sample size (N) for the histology study. (B) Timeline of the 8-week study, including blood collection, craniotomy surgery, weekly treatment dosing, bi-weekly neural recording, and 8-week endpoint procedures for cardiac perfusion and brain collection.

Under sterile conditions, a midline scalp incision (approximately one inch) was made to expose the skull for craniotomy procedures. Hydrogen peroxide was applied to the exposed skull to clean the area by removing blood and connective tissue, creating a dry surface. Butyl cyanoacrylate (VetBond, 3M, Saint Paul, MN) was used to control bleeding, maintain skull dryness, and enhance the adherence of the headcap. Craniotomies for stainless steel bone screws (Stoelting Co., Wood Dale, IL) for ground wires (−1.5 mm anterior/posterior, −1.5 mm lateral to bregma) and reference wires (−5.5 mm anterior/posterior, −1.5 mm lateral to the bregma) were performed using a dental drill with 1.35 mm diameter drill bit. Craniotomies for functional IMEs (+ 2 mm anterior/posterior, + 3 mm lateral to the bregma) and non-functional IMEs (+ 2 mm anterior/posterior and − 3 mm lateral to the bregma) were performed with 1.75 mm diameter drill bit (see schematic in Fig. 1A). Once the dura mater was exposed from drilling, a dura pick was used to reflect the dura. Ground and reference wires on functional IMEs were wrapped and secured on screws before implantation of IMEs. Once the positions were secured, the functional IME was inserted to the motor cortex with 2 mm depth using the stereotaxic frame. Non-functional IMEs were manually implanted at approximately 2 mm depth for improving the sample size (N) for histology study. Excess bone debris and blood from drilling and insertion were then cleaned by applying saline, followed by the application of Kwik-cast to seal the empty space in the craniotomies with implants. Once the Kwik-cast cured, Teets Cold Cure dental cement was applied around all craniotomies to build a headcap for stabilizing all implants and fuse the base to the skull. After the dental cement was cured, 5 – 0 monofilament polypropylene sutures were used to suture the incision site. Cefazolin and meloxicam were subcutaneously administered to the animal as post-operative treatment for infection and pain in a previously reported frequency.

### Dexamethasone Sodium Phosphate Loaded Nanoparticle Manufacture and Characterization

Dexamethasone sodium phosphate loaded platelet-inspired nanoparticles (SPPINDEX) were manufactured with the ‘thin-film rehydration and extrusion’ method adapted from previously established protocols [47, 49, 52, 53]. Briefly, DSPC, cholesterol, DSPE-mPEG(1k), DSPE-PEG(2k)-FMP, DSPE-PEG(2k)-VBP, DSPE-PEG(2k)-CBP and DSPE-Cy5 were homogeneously mixed in 1:1 chloroform:methanol organic solvent at 46.50, 45.00, 6.50, 0.50, 0.25, 0.25 and 1.00 mole percentages, respectively. After the thin lipid film was formed using rotary evaporator, the lipid thin-film was rehydrated with 5% w/v trehalose in 4.05 mg/mL HEPES solution (TH) containing 4 mg dexamethasone sodium phosphate. 10 freeze/thaw cycles were applied to the rehydrated lipid solution with liquid nitrogen and 75°C water bath to maximize drug encapsulation, followed by extrusion with 200 nm then 100 nm polycarbonate membranes using a pneumatic extruder (Evonik, Burnaby, Canada) to form small unilamellar vesicles. Nanoparticle characterizations, including hydrodynamic size distribution, zeta potential, and polydispersity index (PDI) of SPPINDEX, were conducted using dynamic light scattering (DLS) with a Data Litesizer (Anton Paar, Ashland, VA). Zeta potential measurements were performed using an Omega cuvette (Mat. No. 225288, Anton Paar, Ashland, VA) and analyzed under the Smoluchowski approximation with a Henry factor of 1.5. Measurements were conducted using water as the solvent, with the refractive index set to 1.33 and relative permittivity at 78.3. Endotoxin levels in SPPINDEX were evaluated using the Endosafe Limulus Amebocyte Lysate (LAL) kit (#3672151, Charles River Laboratories, Charleston, SC).

### Dexamethasone Sodium Phosphate Loaded Nanoparticles Purification and Encapsulation Efficiency Characterization

Unencapsulated dexamethasone sodium phosphate (DEXSP) was separated from SPPINDEX using a centrifugation method. Freshly manufactured SPPINDEX was transferred to a 10 kDa molecular weight cutoff (MWCO) Amicon filter (Millipore-Sigma, Burlington, MA, UFC801024) in a 15 mL Falcon tube, followed by an initial centrifugation at 3200 × g for 20 minutes. To wash the SPPINDEX trapped on the filters, 0.5 mL of TH solution was added, and a second centrifugation was performed to remove any remaining unencapsulated DEXSP. The mass of the separated unencapsulated DEXSP was quantified via reverse-phase high-performance liquid chromatography (RP-HPLC, Shimadzu). The analysis utilized a C18 reverse-phase column (Shimadzu, Columbia, MD, #220–91199-13) and a UV-vis detector (SPD40V, Shimadzu, Columbia, MD).

For RP-HPLC analysis, unknown encapsulated DEXSP samples and DEXSP standards were prepared using desoximetasone (DES) as an internal standard to account for variations in injection and HPLC system performance. The mobile phase consisted of an isocratic mixture of 32:68 v/v acetonitrile/10 mM phosphate buffer, with an injection volume of 30 μL and a flow rate of 1 mL/min. UV absorbance was measured at 240 nm, with retention times of 3.5 minutes for DEXSP and 22 minutes for DES. Standard calibration curves of the DEXSP-DES peak area ratios and DEXSP-DES concentration ratios were plotted using OriginLab software (OriginLab Corporation, Northampton, MA) to determine unknown DEXSP concentrations. Encapsulation efficiencies were calculated using Eq. (1).

(1) $$Encapsulation\;Efficiency\;(\%)=\frac{\left[\mathrm{Total}\;\mathrm{used}\;\mathrm{drug}\;(mg)-\mathrm{Unencapsulated}\;\mathrm{drug}\;(mg)\right]}{\mathrm{Total}\;\mathrm{used}\;\mathrm{drug}\;(mg)}\times 100\%$$

#### Dexamethasone Sodium Phosphate In Vitro Release

Dialysis membrane (DM) method was employed to characterize the *in vitro* release profile of DEXSP from SPPINDEX [54]. Briefly, 0.5 mL of purified SPPINDEX solution was transferred into a dialysis device (Slide-A-Lyzer^™^ MINI Dialysis Devices, Thermo Fisher Scientific, Waltham, MA) with a 10 kDa MWCO. The device was submerged in 14 mL of 1× PBS within a 15 mL Falcon tube and incubated at 37°C in a shaker set to 70 rpm. At specified time points, 1 mL aliquots were collected from the dialysis medium containing released DEXSP for quantification using RP-HPLC analysis. After each sampling, an equal volume of fresh 1× PBS was added to maintain the dialysis conditions.

### Dexamethasone Sodium Phosphate Loaded Nanoparticles Administration

Twenty-nine catheterized rats were assigned to receive weekly administrations of one of the following treatments: dexamethasone sodium phosphate-loaded platelet-inspired nanoparticles (SPPINDEX, n = 7, 2 mg/kg nanoparticle dosage and 0.3 mg/kg equivalent drug dosage), platelet-inspired nanoparticles (PIN, n = 7, 2 mg/kg nanoparticle dosage), free dexamethasone sodium phosphate (DEXSP, n = 8, 0.3 mg/kg drug dosage), or 5% w/v trehalose in 4.05 mg/mL HEPES solution (TH, n = 7) as diluent control. Administration timeline is shown in Fig. 1B. The infusion method via indwelling vascular catheters was performed according to a previously established protocol [46]. Surgeries to implant vascular catheters were performed by Charles River Labs. Briefly, sterile syringes containing the treatments were connected to external tether tubing with PinPort injectors to access the carotid artery catheters. The treatments were infused towards the aortic arch at a rate of 0.1 mL/min using a syringe pump. Following the infusion, the catheters were maintained by flushing with 500 units/mL heparin to prevent clogging.

### Electrophysiological Recordings

Neural electrophysiology recordings were conducted twice per week for 8 weeks using functional intracortical microelectrodes (IMEs) implanted in rats adapted from previous reported methods [16, 17, 55–58]. The rats were initially anesthetized with 3.5% isoflurane and maintained under surgical anesthesia with 1.5–3% isoflurane during the cleaning of the exposed IME connectors to remove blood and tissue debris using isopropanol. Following this, the rats were transferred into a custom-made acrylic box within a Faraday cage while still under anesthesia. A 16-channel ZIF-Clip Headstage (Tucker-Davis Technologies Inc., Alachua, FL) was attached to both sides of the IME connectors, which were connected to the 32-channel Motorized Commutator System (#ACO32, Tucker-Davis Technologies Inc.). This setup allowed the rats to move freely and minimized motion artifacts. A 16-Channel Medusa Pre-Amp (RA16PA, Tucker-Davis Technologies Inc.) was connected to the commutator to amplify and digitize the analog signals, which were then transmitted to a Bioamp Processor (RZ5, Tucker-Davis Technologies Inc.) via fiber optic connectors for further signal processing. The processed signals were displayed and recorded using Synapse software (Tucker-Davis Technologies Inc.) on a computer. Neural recordings were conducted for 10 minutes after the rats awoke, at a sampling rate of 24.414 kHz with a built-in 300–3000 Hz bandpass filter to capture single-unit activity [59]. After the 10-minute recording session, the rats were disconnected from the headstage and returned to their housing cages.

### Electrophysiological Analysis

Recorded digital neural signals were analyzed using Plexon Offline Sorter (Plexon Inc., Dallas, TX) to identify single units [17, 51]. The data were first referenced to the common median across all 16 channels of the IMEs to eliminate potential artifacts arising from the rats’ movement and common-mode signals. Spikes were detected using − 4σ threshold from the mean of the peak heights. After spike detection, any spikes with amplitudes exceeding ± 500 μV were considered abnormal neuronal signals and were excluded from the analysis. Additionally, spikes detected across more than 14 channels were classified as motion artifacts caused by the rats’ grooming or movement and were removed. Following artifact removal, automatic sorting was performed using the K-Means Scan method to cluster the remaining spikes into individual single units. True positive signal units were manually verified following previously established protocols [58]. The active electrode yield in each treatment group for each week was calculated by dividing the total number of active channels (defined as those with at least one active unit) by the total number of channels (16 channels per IMEs device × number of animals for each treatment in the specific week, excluding channels that never have any active units). The proportion of active channels for each treatment group in each time phase (the first four weeks, W1–4, and the last four weeks, W5–8) was calculated similarly, but by summing the total active and total channels across all weeks in the respective time phase On a weekly basis, the total number of channels (N) was as follows: 107 for SPPINDEX, 104 for PIN, 104 for TH, and 118 for Free DEXSP. For the entire phase, the total number of channels (N) was 428 for SPPINDEX, 416 for PIN, 416 for TH, and 472 for Free DEXSP.

Peak-to-peak voltage (Vpp) was defined as the absolute voltage range from the positive to negative peak of a clustered and verified single unit. Noise was calculated as the root mean square (RMS) of the signal after spike exclusion. Signal-to-noise ratio (SNR) was determined by dividing the Vpp by the noise for each verified single unit. Spike rate was defined as the inverse of the median interspike interval for each verified single unit. Calculated recording parameters were obtained using custom-developed MATLAB software (Mathworks, Natick, MA). Units with a Vpp less than 40 μV were considered non-putative and excluded from further analysis [57]. The calculated recording parameters were grouped into two intervals: the first four weeks of the study (W1–4) and the last four weeks (W5–8). Outliers in the SNR for each unit, across all treatments and phases, were identified using the ROUT method with Q = 5%. Units with verified SNR outliers were excluded from further analysis. Vpp, noise, SNR, and spike rate values for each unit were averaged at the individual channel level for each IME within different treatment groups and time phases, resulting in a single averaged value of Vpp, noise, SNR, and spike rate for each active channel. The total number of units for each active channel was counted and divided by the number of weeks in the specific time phase to calculate the units per active channel. This yielded a single average number of units per active channel for each IME across different treatment groups and time phases, which was used for graph plotting and statistical analysis. For Vpp, noise, SNR, spike rate, and units per active channel data, the sample size (N) represents the total number of active channels for different treatment groups at different phases: 93 for W1–4 SPPINDEX, 51 for W1–4 SPPINDEX, 77 for W1–4 PIN, 43 for W5–8 PIN, 81 for W1–4 TH, 38 for W5–8 TH, 86 for W1–4 Free DEXSP, 30 for W5–8 Free DEXSP.

### Animal Perfusion and Tissue Processing

At the 8-week endpoint, rats were administered an intraperitoneal overdose of ketamine (160 mg/kg) and xylazine (20 mg/kg). Cardiac perfusion and dissection procedures were adapted from a previously established method [60]. In brief, rats were perfused with 600–700 mL of 1× PBS at a ow rate of 50–70 mL/min. Once the liquid flowing from the right atrium was clear, an additional 300–400 mL of 10% neutral buffered formalin solution was perfused. The brains were then dissected and stored in 10% neutral buffered formalin at 4°C for 24 hours, followed by cryoprotection using a 30% w/v sucrose solution containing 0.01% sodium azide. A 10% stepwise increase in sucrose concentration was performed every 24 hours, with two exposures at 30%. The cryoprotected brains were subsequently embedded in Optimal Cutting Temperature (OCT) compound (Scigen Tissue-Plus^™^ O.C.T. Compound, Fisher Scientific, Hampton, NH, USA) and frozen on dry ice, then stored at −80°C. Brain sections were cut transversely at 20 μm thickness using a cryostat (CM1950, Leica, Deer Park, IL).

### Immunohistochemistry, Imaging and Analysis

Immunohistochemistry (IHC) staining was performed on brain slices to visualize and assess neuron loss, microglial activation, astrocyte activation, and blood-brain barrier (BBB) permeability (antibodies listed in Table S1). IHC procedures were adapted from previously reported methods [24, 37, 46, 61, 62]. Brain slices on slides were incubated for 15 minutes in a humidified chamber at room temperature, followed by washing with 1× PBS to remove the O.C.T. compound. The tissues were rehydrated and permeabilized with 1× PBS containing 0.1% Triton for 15 minutes. To prevent non-specific antibody binding, tissues were blocked in a 4% goat serum solution. Primary antibodies were applied and incubated overnight. On the following day, tissues were washed multiple times with 1× PBS containing 0.1% Triton to remove any remaining primary antibodies. Secondary antibodies were then applied for 2 hours. Afterward, a series of washing steps were performed to eliminate any residual secondary antibodies and Triton, followed by the application of DAPI Fluoromount-G mounting medium and coverslips. The slides were dried overnight and stored at 4°C for imaging.

Slides with stained brain slices were imaged using Automatic Slide Scanner Axioscan Z7 (Zeiss Inc., Oberkochen, Germany) with the 20× objective. One slide from each type of staining was used to determine the optimal exposure time. The optimal exposure times were consistent over the entire study. The resulting images were subset around the implantation region and exported as 16-bit TIFF images (10,000 × 10,000 pixels by pixels) using ZEN 3.6 Blue Edition software for fluorescent intensity analysis. A custom Python program, based on SECOND [63], was employed to define implant holes and mask image artifacts. Concentric rings were generated by the program, starting from the outer edge of the implant hole and extending across the image in 50 μm intervals, to measure the average fluorescence intensities in each bin as a function of distance from the hole. Intensities in masked regions due to artifacts were excluded from the measurements. As reported in previous methods [46], average fluorescence intensities for each bin were normalized to the background intensity in the 600–650 μm bin. Since CD68-positive microglia and immunoglobulin G (IgG) are not expected in healthy brain parenchyma, their normalization factors were set to 0, whereas the factor for astrocytes (GFAP), which are normally present, was set to 1.

To count NeuN stained neurons around the implant hole, Cellpose algorithm was used to segment the neurons on NeuN stained slices [64]. As reported in a previous established workflow [65], a specialized cyto3-based model was further trained with at least one stained slide from each treatment group using default parameters. All NeuN images were segmented in batches using the trained model. A custom Python script calculated neuron density from the raw NeuN segmentation masks. Similar to the intensity analysis, neuron density (# of neuron per mm^2^) at each 50 μm bin was calculated by dividing neuron counts by the total area of each bin, starting from the implant site, and normalized to the density in the 600–650 μm bin.

For each IME implant hole, at least three different brain slice depths were analyzed for both the SECOND fluorescent intensity analysis and the Cellpose neuron density analysis. For each treatment group, brain tissues from a minimum of four animals were included in both analyses.

### Blood Collection and Analysis

At weeks 0 (prior to treatment), 4, and 8 (following weekly treatments), rats were anesthetized with 3.5% isoflurane and maintained under a surgical plane of anesthesia (1.5–3% isoflurane). As described in a previously published method [46]. 300 μL of blood was then collected from the tail vein using a capillary blood collection tube (Multivette^®^ 600 Lithium heparin LH, Sarstedt, Germany). After plasma was separated from whole blood by centrifugation, glucose (GLU), creatinine (CREA), and alanine transaminase (ALT) levels were measured in the plasma.

### Statistics

Excel (Microsoft Corporation, Redmon, WA), R Studio 2023.12.0 + 369 (RStudio, PBC, Boston, MA) and GraphPad Prism (Dotmatics, Boston, MA) were used for data analysis, rearrangement and statistical measurements. Custom R scripts were used to rearrange *in vivo* extracellular single-unit recording raw data, including the number of units and active channel yield across different animals, treatments, and weeks. A two-sided proportions z-test was performed using an R-based script to calculate significant statistical differences in active electrode yield between groups on a weekly basis, as well as the proportion of active electrode between and within groups at different study phases. Statistical differences in exported recording parameters (Vpp, noise, SNR, spike rate) were calculated using a mixed-effects model analysis with post hoc Tukey pair-wise comparisons in GraphPad. A one-way ANOVA with post hoc Tukey pair-wise test in GraphPad was used to compare normalized neuron density (NeuN) and normalized fluorescence intensity for astrocyte (GFAP), activated microglia/macrophage (CD68), and Immunoglobulin G (IgG) among treatment groups within each distance interval. The Kruskal-Wallis test with post hoc Dunn’s pair-wise test in GraphPad was conducted to calculate statistical differences in blood parameters and weights measurements. Statistical significance was represented as * = p < 0.05, ** = p < 0.01, *** = p < 0.001 and **** = p < 0.0001 for all statistical analyses in this study. The standard error of the mean (SEM) was used to plot error bars for all box plots.

## Results

### Characterization of SPPINDEX and DEXSP Release Profile

**Figure 2:**
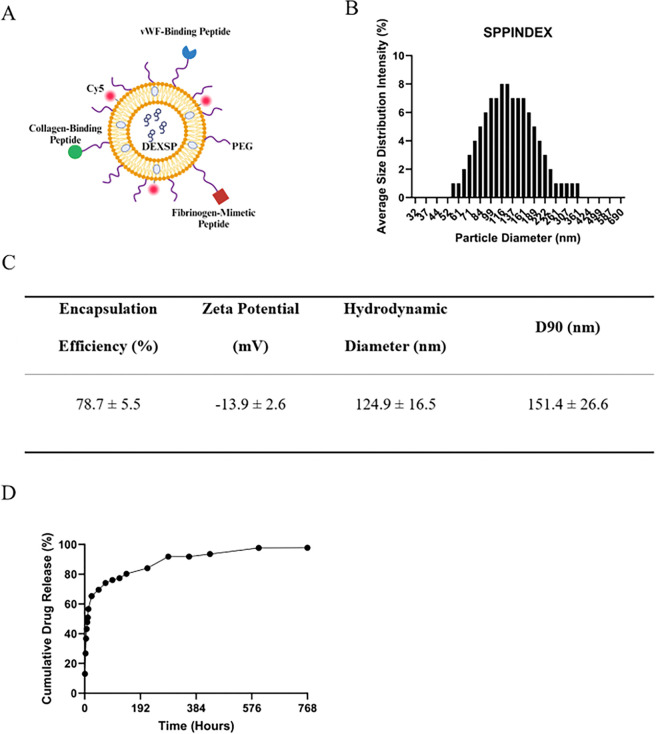
Dexamethasone sodium phosphate loaded platelet-inspired nanoparticles (SPPINDEX) characterization. (A) Schematic of SPPINDEX formulation, created with BioRender. (B) Averaged size distribution of SPPINDEX (over n = 7 manufacture batches). (C) Encapsulation efficiency, zeta potential and hydrodynamic diameter of SPPINDEX in 5% w/v trehalose in 4.05 mg/mL HEPES buffer. (D) *In vitro* release profile of dexamethasone sodium phosphate (DEXSP) from platelet-inspired nanoparticles (PIN) into 1 × PBS solution, showing ~ 65% burst release in 24 hr and ~ 98% release in 24 days.

The schematic of dexamethasone sodium phosphate-loaded platelet-inspired nanoparticles (SPPINDEX) is shown in Fig. 2A. Due to its hydrophilicity, the DEXSP payload is assumed to be predominantly loaded in the aqueous core of the PIN. The encapsulation efficiency of DEXSP in PIN was 78.7 ± 5.5% based on averaged data from seven manufacturing batches. The overall surface charge of SPPINDEX was − 13.9 ± 2.6 mV. The hydrodynamic diameter of SPPINDEX is 124.9 ± 16.5 nm, with 90% of the total particles are smaller than 151.4 ± 26.6 nm (Fig. 2B).

Over time, in an infinite sink model, approximately 65% of DEXSP was released within the first 24 hours (Fig. 2D), with complete release occurring over 24 days into the 1× PBS dialysis solution. The initial burst release of DEXSP from SPPINDEX suggests the necessity of freshly preparing SPPINDEX prior to animal administration. In this study, fresh SPPINDEX was prepared on a weekly basis.

### Therapeutic Efficacy of SPPINDEX on IMEs Recording Performance

**Figure 3:**
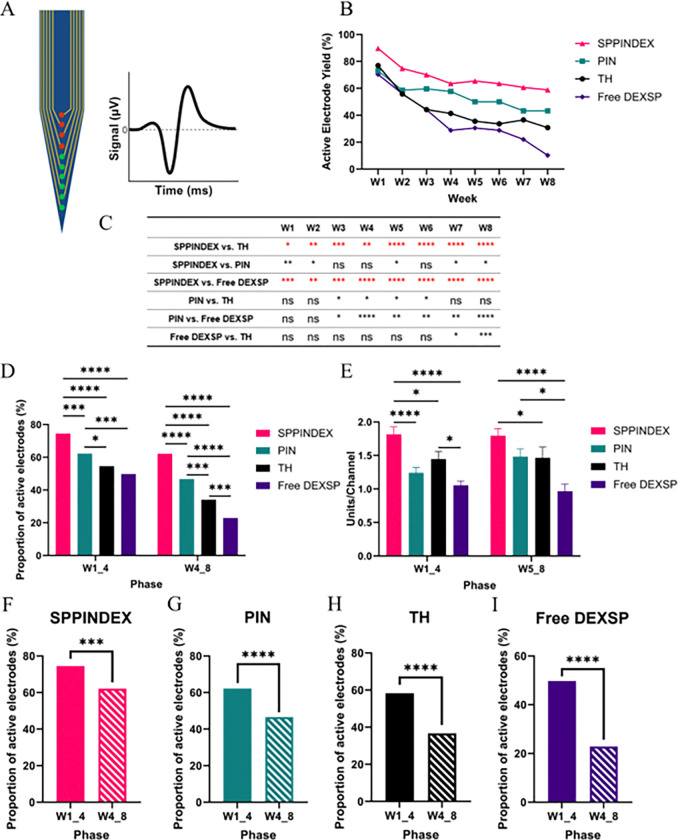
*In vivo* extracellular single-unit electrophysiology recording to evaluate IME recording performance over 8 weeks. (A) Schematic of the IME, showing the active channels (with at least one active unit, green dot) and inactive channels (no active unit, red dots). Active electrode yield (AEY) is defined as the total number of active channels divided by the total number of channels (excluding channels that never have any active units) for each treatment per week. (B) Weekly active electrode yields of different treatments, demonstrating significantly improved recording performance in the SPPINDEX group compared to the PIN, TH and Free DEXSP groups. (C) Statistical comparisons and significant levels for week-by-week actively electrode yields comparisons among groups. (D) Proportion of active electrodes (AEY grouped by phases) for each treatment in the first four weeks (W1_4) and last four weeks (W5_8) of study, indicating that SPPINDEX treatment improved recording performance in both phases. (E) Active units per channel across different treatments and phases, showing significantly better IME single-unit recording ability in the SPPINDEX group compared to all other groups in the first four weeks, with no significant difference compared to the PIN group in the last four weeks. (F-I) Comparisons of the proportion of active electrodes within treatment groups across different phases, indicating a decline in recording performance over time in all groups, with the SPPINDEX group exhibiting significantly less decline compared to other groups.

To acquire and evaluate awake recordings, 16-channel, single-shank functional intracortical microelectrodes (IMEs) were implanted to animals’ brain primary motor cortex. Animals were separated to four cohorts based on the treatments: drug-loaded nanoparticles (SPPINDEX, n = 7), unloaded nanoparticles (PIN, n = 7), free drug (Free DEXSP, n = 8) and diluent control (TH, n = 7). The study timeline is shown in Fig. 1B. Active electrode yield (AEY), calculated to assess recording performance, was determined by conducting biweekly recordings and analyses. It was defined as the percentage of active channels (channels with detected active units) relative to the total potential channels (excluding channels without any detected active units throughout the entire study). Functional IMEs in the SPPINDEX-treated animals demonstrated significantly better performance in terms of AEY compared to the Free DEXSP and TH groups over the 8-week period (see Fig. 3B for trend and Fig. 3C for significance levels). Compared to the PIN group, the AEY of the SPPINDEX group was significantly higher during most weeks, except for weeks 3, 4, and 6.

Active electrode data were grouped to calculate the proportion of active electrodes, which was used to evaluate recording performance across two phases of the study (Fig. 3D): the first four weeks (W1_4) and the last four weeks (W5_8). The SPPINDEX group showed a significantly higher proportion of active electrodes in both phases compared to all other groups (p < 0.001 to PIN, p < 0.0001 to TH, p < 0.0001 to Free DEXSP in W1_4; p < 0.0001 to all other groups in W5_8). The PIN group exhibited a significantly higher proportion of active electrodes in both phases compared to the TH and Free DEXSP groups (p < 0.05 to TH, p < 0.001 to Free DEXSP in W1_4; p < 0.001 to TH, p < 0.0001 to Free DEXSP in W5_8). No significant differences in the proportion of active electrodes were observed between the TH and Free DEXSP groups during the first week of the study. However, in the last four weeks, the proportion of active electrodes in the Free DEXSP group was significantly lower than in the TH group (p < 0.001). Within each treatment group, the proportion of active electrodes significantly decreased in the last four weeks of the study (p < 0.001 in SPPINDEX, p < 0.0001 in PIN, p < 0.0001 in TH, p < 0.0001 in Free DEXSP), indicating a decline in IME recording performance over time (Fig. 3F – 3I). Nevertheless, the SPPINDEX group exhibited a 17% decline in active electrode yield, which was less pronounced compared to the declines in the other groups: 25% for PIN, 37% for TH, and 54% for Free DEXSP.

Active units per channel were calculated to assess the IMEs’ ability to record single units (Fig. 3E). During the first four weeks of the study, SPPINDEX treatment significantly improved active units per channel compared to all other groups (p < 0.0001 to PIN, p < 0.05 to TH, p < 0.0001 to Free DEXSP). In the last four weeks, SPPINDEX maintained significantly higher active units per channel compared to the TH and Free DEXSP groups (p < 0.05 to TH, p < 0.0001 to Free DEXSP), but not compared to the PIN group. Within each treatment group, no significant differences were observed between the different phases of the study for any treatment group.

**Figure 4:**
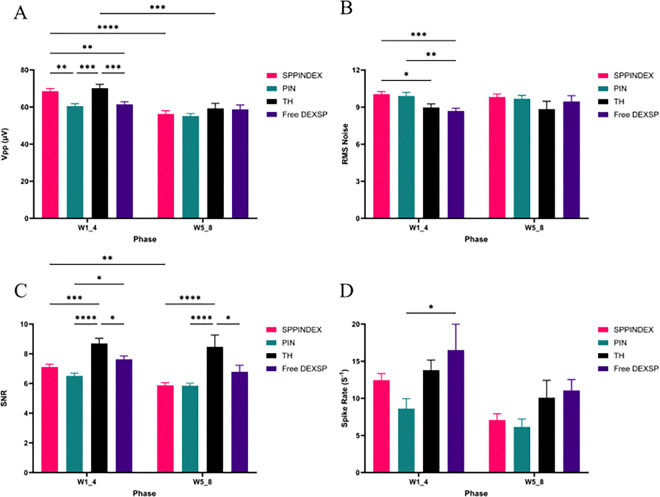
Exported *in vivo* extracellular electrophysiology metrics. (A) peak-to-peak voltage of signals (Vpp) is significantly higher in the SPPINDEX group compared to the PIN and Free DEXSP groups during the first four weeks of the study, no significant difference between the SPPINDEX and TH groups. (B) Significantly higher noise levels were observed in the SPPINDEX group compared to the TH and Free DEXSP groups during the first four weeks. (C) Signal-to-noise ratio (SNR) was significantly lower in the SPPINDEX group compared to the TH group in both study phases. (D) No significant difference in spike rate was observed between SPPINDEX and TH groups in either phase.

Peak-to-peak voltage (Vpp) was significantly higher in the SPPINDEX group compared to the PIN and Free DEXSP groups (p < 0.01 to PIN and Free DEXSP) during the first four weeks of the study, but there was no significant difference between the SPPINDEX and TH groups, suggesting that SPPINDEX treatment may not substantially influence signal intensity (Fig. 4A). In the last four weeks of the study, no significant differences in Vpp were observed among the groups. Noise levels in the SPPINDEX group were significantly higher compared to the TH and Free DEXSP groups (p < 0.05 to TH, p < 0.001 to Free DEXSP) during the first four weeks, indicating a potential increase in noise associated with SPPINDEX treatment during the early weeks (Fig. 4B). However, this effect did not persist in the later weeks of the study. Although the signal-to-noise ratio (SNR) was significantly lower in the SPPINDEX group compared to the TH group (p < 0.001 in W1_4, p < 0.0001 in W5_8) for both phases of the study, the mean SNR remained above 5 for all treatments, demonstrating high-quality recordings across all groups (Fig. 4C). There were no significant differences in spike rate between the SPPINDEX group and the other groups (Fig. 4D).

### Therapeutic Effects of SPPINDEX on Neuron Health and Density

**Figure 5:**
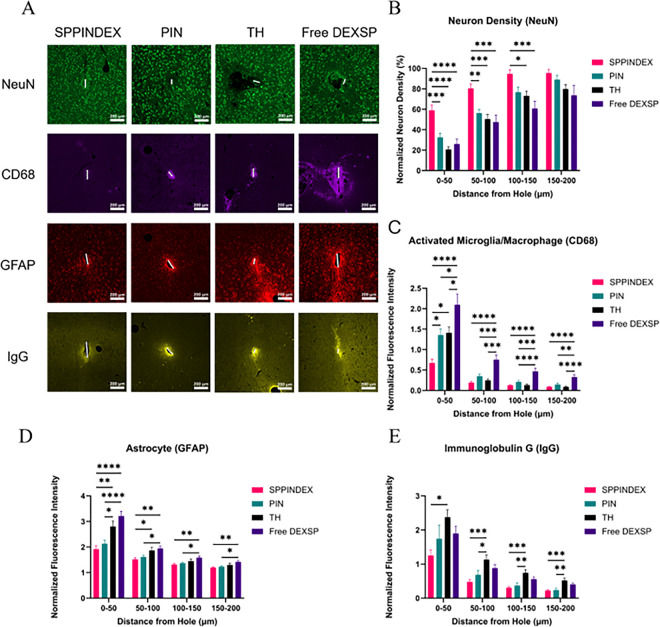
Immunofluorescent intensities of neurons (NeuN), activated microglia/macrophages (CD68), astrocyte (GFAP) and immunoglobulin G (IgG) near implant sites at 8-week endpoint. (A) Representative images at the 8-week endpoint. Images were false colored. Scale bars = 200 μm. (B) Normalized neuron density as a function of distance from the interface of microelectrodes (neuron counts in each bin normalized to neuron counts in the 600–650 μm bin). The SPPINDEX group exhibited significantly higher normalized neuron densities compared to all other treatments up to 100 μm from the interface. (C-E) Normalized immunofluorescent intensity of CD68, GFAP, and IgG as a function of distance from the interface of microelectrodes. C-E) Normalized immunofluorescent intensity of CD68, GFAP, and IgG as a function of distance from the microelectrode interface. (C) CD68 intensity was significantly lower in the SPPINDEX group compared to all other groups up to 50 μm from the implant site. (D) GFAP intensity was significantly lower in the SPPINDEX group compared to the TH and Free DEXSP groups up to 100 μm. (E) IgG intensity was significantly lower in the SPPINDEX group compared to the TH group up to 600 μm (see Table S2 – S5 for full statistical comparisons across different bins for different biomarkers).

The acute death of resident neurons following IMEs insertion, along with secondary neuron death caused by the insertion-induced neuroinflammatory response, severely reduces healthy neuron density in the motor cortex, leading to a scarcity of neuronal activity recording resources. Neuronal populations were assessed using neuronal nuclei (NeuN) staining. To evaluate the therapeutic effects of SPPINDEX on neuronal health and viability around the insertion site, brain tissues from all groups were stained with NeuN at the 8-week endpoint. Weekly systemic administration of SPPINDEX over 8 weeks significantly attenuated the reduction in neuron density up to 150 μm from the implant site compared to the PIN, TH and Free DEXSP groups (Fig. 5B). Specifically, the SPPINDEX group (59% neuron density normalized to neuron counts in 600–650 μm distant area of tissue) exhibited a 186% increase in neuron densities compared to the TH group (21% normalized neuron density) at the 0–50 μm bin from the implant site, indicating that SPPINDEX treatment significantly enhances the availability of healthy neurons for electrophysiology neural signal recording near the IME interface. This increase in neuron density likely contributes to the improved recording performance observed with SPPINDEX treatment.

### Therapeutic Effects of SPPINDEX on Neuroinflammation and BBB Permeability

In addition to the acute neuron death caused by the IME insertion, microglia and macrophages are also activated and recruited to the implant site through both DAMPs (cell debris from neuron death) and PAMPs (influx of blood-derived proteins) signaling pathways [66–69]. Chronic activation and recruitment of microglia and macrophages induces self-perpetuating neuroinflammation and cell encapsulation on IMEs interface, leading to chronic failure of IME recording performance. At the 8-week endpoint, activated and recruited microglia intensity (CD68) was found significantly lower up to 50 μm from the implant site in animals treated with SPPINDEX in comparison to those treated with PIN, TH and Free DEXSP (Fig. 5C). Animals treated with weekly SPPINDEX over 8 weeks had significantly lower expression of astrocyte (GFAP) up to 100 μm from the implant site in comparison to the TH and Free DEXSP groups (Fig. 5D). Animals treated with weekly PIN over 8 weeks also showed significantly lower intensity of astrocyte up to 50 μm from the implant site in comparison to the TH and Free DEXSP groups Free DEXSP groups showed the significantly highest activated microglia expression levels near the implant site.

BBB permeability was evaluated by staining immunoglobulin G (IgG) to assess the potential resealing function of SPPINDEX and PIN groups. IgG is a classic biomarker for BBB leakage, as it is a blood serum protein and not commonly found in the brain parenchyma. In the SPPINDEX group, IgG intensity was significantly reduced up to 550 μm from the implant site in comparison to the TH group (Fig. 5E, significant levels after 200 μm shown in Table S5). Similarly, animals treated with PIN also had significantly lower IgG intensity starting from 50 μm up to 600 μm from the implant site compared to the TH group (Table S5). The significantly reduced IgG levels in both the SPPINDEX and PIN groups suggest that the nanoparticles may facilitate blood-brain barrier (BBB) resealing and promote hemostasis at sites of IME insertion-induced microhemorrhage.

### Side Effects of SPPINDEX on Metabolic Homeostasis and Organ Functions

**Figure 6:**
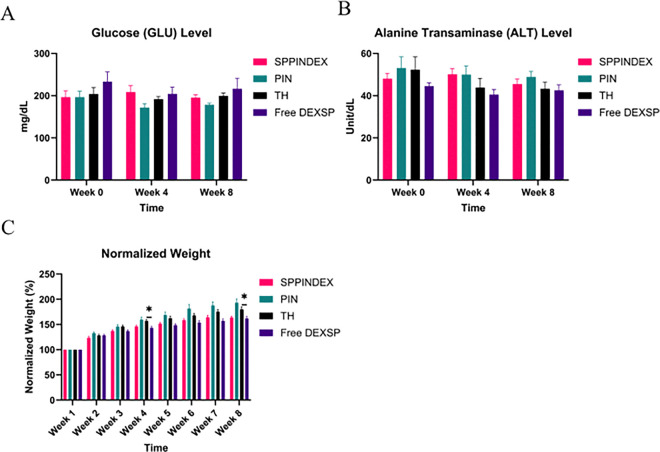
Safety evaluations of weekly treatments over 8 weeks. (A) Glucose (GLU) levels showed no significant differences among all groups at week 0, week 4, and week 8. (B) Alanine transaminase (ALT) levels did not significantly vary among groups at week 0, week 4, and week 8, indicating no liver dysfunction. (C) Normalized body weights in the Free DEXSP group were significantly lower compared to the TH group at week 4 and week 8, suggesting potential adverse effects on body weights from non-selective DEXSP treatment.

Long-term systematic dexamethasone treatment might induce multiple side effects including abnormally high glucose level, weight loss, and bone mineral density (BMD) loss in rodent model [45]. To ensure weekly dosage of SPPINDEX does not induce severe side effects on metabolism, liver and kidney functions in rats, we measured glucose levels, alanine transaminase (ALT) levels, and creatinine (CREA) levels at week 0, week 4 and week 8, weights weekly. No significant change on glucose levels and weights in the animals treated with SPPINDEX in comparison to those treated with control TH (Fig. 6A, Fig. 6C). Weights in the animals were significantly reduced in Free DEXSP group compared to the TH at week 4 and week 8, suggestion temporary effects on metabolism of animals from weekly repeated Free DEXSP treatment. ALT levels were also not significantly abnormal in the SPPINDEX group in comparison to the control TH group (Fig. 6B). Most CREA levels were below detection limits, indicating minimal fluctuations of kidney functions among all groups (Table S6), and therefore the data was omitted from plotting in Fig. 6.

## Discussion

Intracortical microelectrodes are widely used in neuroscience research and BMI systems for restoring the loss of motor function in patients with limb loss and spinal cord injury [70–74]. However, electrophysiological recording quality significantly declines within weeks after implantation. This decline is primarily due to a self-perpetuating neuroinflammatory response triggered by IME insertion-induced tissue damage and the infiltration of blood-derived proteins and pathogens into the brain parenchyma through a compromised blood-brain barrier (BBB) [75–80].

IME insertion-induced bleeding and tissue damage is unavoidable with current devices, prompting extensive research into novel therapies aimed at disrupting insertion-induced neuroinflammation in the brain parenchyma. This neuroinflammation is characterized by the infiltration of microglia and astrocytes at the implant site, accompanied by the release of proinflammatory cytokines such as TNF-alpha and IL-1 beta, as well as cytotoxic factors like reactive nitrogen species (RNS), reactive oxygen species (ROS), and nitric oxide (NO), causing secondary cellular and tissue damage (neuron death) surrounding the implant [78, 80].

Gaire *et al.* investigated the effects of acute systemic administration of DEX and found that it might not improve chronic recording quality due to off-target drug delivery. Additionally, long-term systemic dosing of DEX is contraindicated, as it can cause numerous side-effects and complications [38]. In contrast, Zhong *et al.* explored local delivery of DEX by coating microelectrodes with DEX-encapsulated nitrocellulose prior to insertion, aiming to avoid off-target effects and the safety risks associated with systemic administration [39]. Although this approach significantly reduced neuroinflammation and improved signal recording quality, it was limited by a single administration and depletion of the drug, making it inadequate for long-term treatment. Both systemic and local delivery of free DEX show promise for mitigating insertion-induced neuroinflammation, but each has its own limitations. There are limited studies focused on preventing the triggers of neuroinflammation, such as enhancing hemostasis to manage insertion-induced microhemorrhages and resealing the compromised blood-brain barrier (BBB) to reduce the infiltration of foreign substances into the brain parenchyma.

The physical damage to cells and tissues in the brain parenchyma caused by IME insertion is inevitable and partially induces a neuroinflammatory response through the DAMPs signaling pathway [66, 81]. Key triggers of neuroinflammation, such as the influx of blood-derived proteins and pathogens into the brain parenchyma through compromised blood vessels and the blood-brain barrier (BBB), can potentially be targeted and mitigated through the application of biomaterial-based hemostatic interventions. Biomaterials designed to enhance hemostasis in brain trauma and to repair brain tissue and blood vasculature show promise for reducing intracerebral hemorrhage, thereby preventing secondary injuries such as inflammation and cerebral edema [82]. Therefore, the principal hypothesis of the current study is that systemic administration of platelet-inspired nanoparticles (PIN) can promote hemostasis at the site of insertion-induced microhemorrhages and reseal the compromised BBB. This would reduce the infiltration of blood-derived proteins and pathogens into the brain parenchyma. Additionally, activated platelets and coagulation factors such as von Willebrand factor (vWF), collagen have been found to persist near the implant site and are associated with neuroinflammation for up to 8 weeks [37]. Localization and retention of PINs at the implant site could also enable targeted delivery of anti-inflammatory agents, such as dexamethasone sodium phosphate, further enhancing the attenuation of self-perpetuating neuroinflammation beyond their hemostatic function. This combination therapy, using anti-inflammatory drug-encapsulated hemostatic nanoparticles, has the potential to improve long-term neural recording performance.

In this study, we demonstrated that the decline in active electrode yield over time was significantly attenuated in animals treated with SPPINDEX compared to those receiving other treatments (PIN, TH, and Free DEXSP) throughout the 8-week study period. Specifically, the proportion of active electrodes was approximately 36% higher in the first half of the study and 82% higher in the second half with SPPINDEX treatment compared to TH treatment, indicating significantly improved neural recording performance in animals receiving weekly SPPINDEX administration. Significantly lower IgG levels around the implant sites were observed in the SPPINDEX group, suggesting that the improved recording performance may be due to reduced infiltration of blood-derived substances into the brain parenchyma. This reduction in blood-derived substance infiltration resulted in less activation and recruitment of microglia near the implant site in the SPPINDEX group compared to the TH and Free DEXSP groups, further supporting our hypothesis that the resealing and hemostatic properties of nanoparticles can mitigate the influx of blood-derived proteins, thereby reducing cells infiltration and neuroinflammation around the implant site.

The significant improvement in active electrode yield observed in the PIN-only group compared to the TH and Free DEXSP groups validates that the nanoparticle itself, even without drug loading, can enhance recording performance by resealing the compromised BBB and inhibiting the influx of blood-derived proteins, as seen in histological analysis. This reduced influx also attenuated astrocyte infiltration near the implant site compared to both the TH and Free DEXSP groups. The significant reduction in IgG levels in both the SPPINDEX and PIN groups, with no significant difference between these groups, provides further evidence that the PIN vehicle alone has the potential to reduce the influx of blood-derived substances by resealing the ruptured BBB.

Surprisingly, the Free DEXSP group exhibited significantly worse recording performance compared to the negative control (TH group), consistent with findings from previous studies on the long-term therapeutic efficacy of systemic DEX administration for treating neuroinflammation and improving IME electrophysiology recording quality [38, 41]. Chen *et al.* reported that a single, non-targeted dose of dexamethasone promoted wound healing in septic mice with pathogenic inflammation but delayed wound healing in mice without pathogenic inflammation[83]. This observation may explain the exacerbated neuroinflammation observed in the Free DEXSP group in our study, as healthy rats without pathogenic inflammation were used. The poorer recording performance in the Free DEXSP group suggests the importance of targeted drug delivery. This study demonstrates that targeted delivery using PIN was successfully achieved.

For the SPPINDEX treatment, in addition to initially disrupting self-perpetuating neuroinflammation by reducing the invasion of foreign substances into the brain parenchyma, dexamethasone sodium phosphate (DEXSP) was potentially released into the brain parenchyma and absorbed by activated microglia near the implant site via the injury-targeted delivery platform (PIN). By binding to the glucocorticoid receptor (GC) on microglia, DEXSP can internalize into cytoplasm, where it binds to the cytokine-activated NF-κB complex (p50 and p65 subunits) and promotes expression of NF-kB inhibitor IκBα. This inhibits the activated NF-κB complex and reduces the expression of proinflammatory cytokines [84–86]. The attenuation of proinflammatory cytokines promotes an anti-inflammatory microenvironment near the implant site, potentially reducing the secondary neuronal damage and astrocytes recruitment, further disrupting the self-perpetuating neuroinflammation induced by IME insertion. This dual disruption of neuroinflammation fosters a less proinflammatory environment, leading to improved neuronal health, increased neuron density, reduced infiltration of activated microglia, and the most significant improvement in neural recording performance compared to all other treatments.

It is important to note that this study did not assess proinflammatory cytokine levels in brain tissue to investigate the mechanism of SPPINDEX’s therapeutical effects on promoting anti-inflammatory microenvironment near the implant site. This limitation was due to technical challenges in histologically staining extracellular cytokines on formalin-perfused tissues. To address this issue, future studies can focus on using spatial transcriptomics analysis on brain slices to evaluate the neuroinflammatory-specific DNA/RNA expression levels using NanoString for bulk tissue analysis at the microelectrode-tissue interface. Sydney *et al*.[87] demonstrated the successful evaluation of over 800 neuroinflammatory-specific DNA/RND expression levels including IL-1B and TNF-alpha, two common proinflammatory cytokines produced by activated microglia.

Dexamethasone sodium phosphate is a synthetic glucocorticoid. High dose or long-term administration of DEXSP might develop several side effects such as hyperglycemia and weight loss due to its non-selective action in animals [45]. Additionally, we previously characterized the biodistribution of PINs in the livers and kidneys of rats, raising concerns about potential organ dysfunction with long-term PIN dosing. No significant differences in glucose levels were observed among groups at weeks 0, 4, and 8, indicating that hyperglycemia did not occur in the SPPINDEX group. For normalized animal weights with different treatment over 8 weeks, only the Free DEXSP group showed significantly lower weights compared to the TH group at week 4, suggesting that non-selective Free DEXSP administration may affect weight but not persist in later weeks. There were no significant differences in normalized weight between the SPPINDEX and TH groups, indicating that targeted delivery of DEXSP with PIN may reduce the long-term side effects of DEXSP on body weight compared to Free DEXSP treatment. Alanine transaminase (ALT) levels, a typical enzyme marker for liver function, did not show significant fluctuations among groups. Combined with low levels of creatinine (a kidney waste) among all group, weekly treatments of SPPINDEX, PIN and Free DEXSP didn’t induce dysfunctional liver and kidney. Overall, DEXSP encapsulated in PIN did not significantly impact metabolism, liver and kidney function in rats during the 8-week repeated dosing study, indicating the safety of this treatment approach.

The clinical translatability of intra-arterial administration is limited due to its unexpected risks, vascular and neural toxicity [88], particularly with repeated weekly dosing. The initial intention to use intra-arterial injection with pre-implanted catheters in this study was to avoid complications such as phlebitis from weekly tail vein injections, which could affect the quality of drug delivery. Future studies will focus on optimizing SPPINDEX dosing by reducing the treatment duration from 8 weeks of weekly administration to 4 weeks while evaluating whether the recording performance can be sustained up to 8 weeks and even 16 weeks, using a larger animal model such as rabbits via intravenous injection. This approach could potentially enhance the clinical translatability of systemic SPPINDEX treatment for reducing neuroinflammation and improving neural recording performance.

## Conclusion

Dexamethasone sodium phosphate (DEXSP) is widely used to develop anti-inflammatory therapies aimed at mitigating IME insertion-induced neuroinflammation and enhancing electrophysiological recording performance through either systemic or local delivery, each with its own limitations. Our study demonstrates that treatment with DEXSP-loaded platelet-inspired nanoparticles significantly improves active electrode yield for up to 8 weeks. This enhanced neural recording performance is likely due to the initial prevention of blood-derived protein influx, facilitated by the resealing of the leaky blood-brain barrier (BBB) through the hemostatic function of platelet-inspired nanoparticles. Furthermore, the targeted delivery of DEXSP to the IME implant interface using injury-targeting platelet-inspired nanoparticles further attenuates IME insertion-induced neuroinflammation by disrupting the signaling pathways involved in the neuroinflammatory cascade. The combination of hemostasis-promoting platelet-inspired nanoparticles and targeted delivery of the anti-inflammatory drug DEXSP to the IME implant site significantly reduces neuroinflammation and improves the neural recording performance of IMEs. Future studies could investigate the specific mechanisms by which targeted DEXSP delivery interrupts IME-induced perpetuating neuroinflammation and investigate alternative administration routes for SPPINDEX treatment to improve clinical translatability.

## Data Availability

Data will be made available on Shofflab Github repository.

## Highlights

- We developed dexamethasone-loaded platelet-inspired nanoparticles (SPPINDEX).
- We evaluated intracortical microelectrode (IME) recording performance for 8 weeks.
- SPPINDEX treatment improved IME recording performance up to 8 weeks in rats.
- SPPINDEX treatment increased the neuron density near the IME implant site.
- Our treatment also reduced activated microglia, astrocyte and BBB permeability.

## References

[1] Chestek C.A., Gilja V., Nuyujukian P., Foster J.D., Fan J.M., Kaufman M.T., Churchland M.M., Rivera-Alvidrez Z., Cunningham J.P., Ryu S.I., Shenoy K. V., Long-term stability of neural prosthetic control signals from silicon cortical arrays in rhesus macaque motor cortex, in: J Neural Eng, 2011. 10.1088/1741-2560/8/4/045005. PMC3644617 21775782

[2] Ortiz-Rosario A., Adeli H., Brain-computer interface technologies: From signal to action, Rev Neurosci 24 (2013) 537–552. 10.1515/revneuro-2013-0032. 24077619

[3] Chen R., Canales A., Anikeeva P., Neural recording and modulation technologies, Nat Rev Mater 2 (2017). 10.1038/natrevmats.2016.93. PMC6707077 31448131

[4] Jorfi M., Skousen J.L., Weder C., Capadona J.R., Progress towards biocompatible intracortical microelectrodes for neural interfacing applications, J Neural Eng 12 (2015). 10.1088/1741-2560/12/1/011001. PMC4428498 25460808

[5] Pancrazio J.J., Peckham P.H., Neuroprosthetic devices: How far are we from recovering movement in paralyzed patients?, Expert Rev Neurother 9 (2009) 427–430. 10.1586/ern.09.12. 19344294 PMC2685465

[6] Ajiboye A.B., Willett F.R., Young D.R., Memberg W.D., Murphy B.A., Miller J.P., Walter B.L., Sweet J.A., Hoyen H.A., Keith M.W., Peckham P.H., Simeral J.D., Donoghue J.P., Hochberg L.R., Kirsch R.F., Restoration of reaching and grasping movements through brain-controlled muscle stimulation in a person with tetraplegia: a proof-of-concept demonstration, The Lancet 389 (2017) 1821–1830. 10.1016/S0140-6736(17)30601-3. PMC5516547 28363483

[7] Hochberg L.R., Serruya M.D., Friehs G.M., Mukand J.A., Saleh M., Caplan A.H., Branner A., Chen D., Penn R.D., Donoghue J.P., Neuronal ensemble control of prosthetic devices by a human with tetraplegia, Nature 442 (2006) 164–171. 10.1038/nature04970. 16838014

[8] Fatima N., Shuaib A., Saqqur M., Intra-cortical brain-machine interfaces for controlling upper-limb powered muscle and robotic systems in spinal cord injury, Clin Neurol Neurosurg 196 (2020). 10.1016/j.clineuro.2020.106069. 32682223

[9] Simeral J.D., Kim S.P., Black M.J., Donoghue J.P., Hochberg L.R., Neural control of cursor trajectory and click by a human with tetraplegia 1000 days after implant of an intracortical microelectrode array, in: J Neural Eng, 2011. 10.1088/1741-2560/8/2/025027. PMC3715131 21436513

[10] Vilela M., Hochberg L.R., Applications of brain-computer interfaces to the control of robotic and prosthetic arms, in: Handb Clin Neurol, Elsevier B.V., 2020: pp. 87–99. 10.1016/B978-0-444-63934-9.00008-1. 32164870

[11] Colachis S.C., Dunlap C.F., Annetta N. V., Tamrakar S.M., Bockbrader M.A., Friedenberg D.A., Long-term intracortical microelectrode array performance in a human: A 5 year retrospective analysis, J Neural Eng 18 (2021). 10.1088/1741-2552/ac1add. 34352736

[12] Barrese J.C., Rao N., Paroo K., Triebwasser C., Vargas-Irwin C., Franquemont L., Donoghue J.P., Failure mode analysis of silicon-based intracortical microelectrode arrays in non-human primates, J Neural Eng 10 (2013). 10.1088/1741-2560/10/6/066014. PMC4868924 24216311

[13] Bedell H.W., Hermann J.K., Ravikumar M., Lin S., Rein A., Li X., Molinich E., Smith P.D., Selkirk S.M., Miller R.H., Sidik S., Taylor D.M., Capadona J.R., Targeting CD14 on blood derived cells improves intracortical microelectrode performance, Biomaterials 163 (2018) 163–173. 10.1016/j.biomaterials.2018.02.014. 29471127 PMC5841759

[14] Michelson N.J., Vazquez A.L., Eles J.R., Salatino J.W., Purcell E.K., Williams J.J., Cui X.T., Kozai T.D.Y., Multi-scale, multi-modal analysis uncovers complex relationship at the brain tissue-implant neural interface: New emphasis on the biological interface, J Neural Eng 15 (2018). 10.1088/1741-2552/aa9dae. PMC5967409 29182149

[15] Saxena T., Karumbaiah L., Gaupp E.A., Patkar R., Patil K., Betancur M., Stanley G.B., Bellamkonda R. V., The impact of chronic blood-brain barrier breach on intracortical electrode function, Biomaterials 34 (2013) 4703–4713. 10.1016/j.biomaterials.2013.03.007. 23562053

[16] Kim Y., Ereifej E.S., Schwartzman W.E., Meade S.M., Chen K., Rayyan J., Feng H., Aluri V., Mueller N.N., Bhambra R., Bhambra S., Taylor D.M., Capadona J.R., Investigation of the feasibility of ventricular delivery of resveratrol to the microelectrode tissue interface, Micromachines (Basel) 12 (2021). 10.3390/mi12121446. PMC8708660 34945296

[17] Hoeferlin G.F., Bajwa T., Olivares H., Zhang J., Druschel L.N., Sturgill B.S., Sobota M., Boucher P., Duncan J., Hernandez-Reynoso A.G., Cogan S.F., Pancrazio J.J., Capadona J.R., Antioxidant Dimethyl Fumarate Temporarily but Not Chronically Improves Intracortical Microelectrode Performance, Micromachines (Basel) 14 (2023). 10.3390/mi14101902. PMC10609067 37893339

[18] Polikov V.S., Tresco P.A., Reichert W.M., Response of brain tissue to chronically implanted neural electrodes, J Neurosci Methods 148 (2005) 1–18. 10.1016/j.jneumeth.2005.08.015. 16198003

[19] Gulino M., Kim D., Pané S., Santos S.D., Pêgo A.P., Tissue response to neural implants: The use of model systems toward new design solutions of implantable microelectrodes, Front Neurosci 13 (2019). 10.3389/fnins.2019.00689. PMC6624471 31333407

[20] Bjornsson C.S., Oh S.J., Al-Kofahi Y.A., Lim Y.J., Smith K.L., Turner J.N., De S., Roysam B., Shain W., Kim S.J., Effects of insertion conditions on tissue strain and vascular damage during neuroprosthetic device insertion, J Neural Eng 3 (2006) 196–207. 10.1088/1741-2560/3/3/002. 16921203

[21] Hoeferlin G.F., Menendez D.M., Krebs O.K., Capadona J.R., Shoffstall A.J., Assessment of Thermal Damage from Robot-Drilled Craniotomy for Cranial Window Surgery in Mice, Journal of Visualized Experiments 2022 (2022). 10.3791/64188. 36440896

[22] Shoffstall A.J., Paiz J.E., Miller D.M., Rial G.M., Willis M.T., Menendez D.M., Hostler S.R., Capadona J.R., Potential for thermal damage to the blood-brain barrier during craniotomy: Implications for intracortical recording microelectrodes, J Neural Eng 15 (2018). 10.1088/1741-2552/aa9f32. PMC6482047 29205169

[23] Bennett C., Mohammed F., Álvarez-Ciara A., Nguyen M.A., Dietrich W.D., Rajguru S.M., Streit W.J., Prasad A., Neuroinflammation, oxidative stress, and blood-brain barrier (BBB) disruption in acute Utah electrode array implants and the effect of deferoxamine as an iron chelator on acute foreign body response, Biomaterials 188 (2019) 144–159. 10.1016/j.biomaterials.2018.09.040. 30343257 PMC6300159

[24] Potter K.A., Buck A.C., Self W.K., Capadona J.R., Stab injury and device implantation within the brain results in inversely multiphasic neuroinflammatory and neurodegenerative responses, J Neural Eng 9 (2012). 10.1088/1741-2560/9/4/046020. 22832283

[25] Bennett C., Samikkannu M., Mohammed F., Dietrich W.D., Rajguru S.M., Prasad A., Blood brain barrier (BBB)-disruption in intracortical silicon microelectrode implants, Biomaterials 164 (2018) 1–10. 10.1016/j.biomaterials.2018.02.036. 29477707 PMC5895107

[26] Ravikumar M., Sunil S., Black J., Barkauskas D.S., Haung A.Y., Miller R.H., Selkirk S.M., Capadona J.R., The roles of blood-derived macrophages and resident microglia in the neuroinflammatory response to implanted Intracortical microelectrodes, Biomaterials 35 (2014) 8049–8064. 10.1016/j.biomaterials.2014.05.084. 24973296 PMC4169074

[27] Kozai T.D.Y., Marzullo T.C., Hooi F., Langhals N.B., Majewska A.K., Brown E.B., Kipke D.R., Reduction of neurovascular damage resulting from microelectrode insertion into the cerebral cortex using in vivo two-photon mapping, J Neural Eng 7 (2010). 10.1088/1741-2560/7/4/046011. PMC3164482 20644246

[28] Potter K.A., Buck A.C., Self W.K., Callanan M.E., Sunil S., Capadona J.R., The effect of resveratrol on neurodegeneration and blood brain barrier stability surrounding intracortical microelectrodes, Biomaterials 34 (2013) 7001–7015. 10.1016/j.biomaterials.2013.05.035. 23791503

[29] Potter-Baker K.A., Nguyen J.K., Kovach K.M., Gitomer M.M., Srail T.W., Stewart W.G., Skousen J.L., Capadona J.R., Development of superoxide dismutase mimetic surfaces to reduce accumulation of reactive oxygen species for neural interfacing applications, J Mater Chem B 2 (2014) 2248–2258. 10.1039/c4tb00125g. 25132966 PMC4131700

[30] Kozai T.D.Y., Li X., Bodily L.M., Caparosa E.M., Zenonos G.A., Carlisle D.L., Friedlander R.M., Cui X.T., Effects of caspase-1 knockout on chronic neural recording quality and longevity: Insight into cellular and molecular mechanisms of the reactive tissue response, Biomaterials 35 (2014) 9620–9634. 10.1016/j.biomaterials.2014.08.006. 25176060 PMC4174599

[31] Simpson D.S.A., Oliver P.L., Ros generation in microglia: Understanding oxidative stress and inflammation in neurodegenerative disease, Antioxidants 9 (2020) 1–27. 10.3390/antiox9080743. PMC7463655 32823544

[32] Ward M.P., Rajdev P., Ellison C., Irazoqui P.P., Toward a comparison of microelectrodes for acute and chronic recordings, Brain Res 1282 (2009) 183–200. 10.1016/j.brainres.2009.05.052. 19486899

[33] Biran R., Martin D.C., Tresco P.A., The brain tissue response to implanted silicon microelectrode arrays is increased when the device is tethered to the skull, J Biomed Mater Res A 82 (2007) 169–178. 10.1002/jbm.a.31138. 17266019

[34] Salatino J.W., Ludwig K.A., Kozai T.D.Y., Purcell E.K., Glial responses to implanted electrodes in the brain, Nat Biomed Eng 1 (2017) 862–877. 10.1038/s41551-017-0154-1. 30505625 PMC6261524

[35] Mercanzini A., Colin P., Bensadoun J.C., Bertsch A., Renaud P., In vivo electrical impedance spectroscopy of tissue reaction to microelectrode arrays, IEEE Trans Biomed Eng 56 (2009) 1909–1918. 10.1109/TBME.2009.2018457. 19362904

[36] Prasad A., Sanchez J.C., Quantifying long-term microelectrode array functionality using chronic in vivo impedance testing, J Neural Eng 9 (2012). 10.1088/1741-2560/9/2/026028. 22442134

[37] Lam D. V., Javadekar A., Patil N., Yu M., Li L., Menendez D.M., Sen Gupta A., Capadona J.R., Shoffstall A.J., Platelets and hemostatic proteins are co-localized with chronic neuroinflammation surrounding implanted intracortical microelectrodes, Acta Biomater 166 (2023) 278–290. 10.1016/j.actbio.2023.05.004. 37211307 PMC10330779

[38] Gaire J., Lee H.C., Hilborn N., Ward R., Regan M., Otto K.J., The role of inflammation on the functionality of intracortical microelectrodes, J Neural Eng 15 (2018). 10.1088/1741-2552/aae4b6. 30260321

[39] Zhong Y., Bellamkonda R. V., Dexamethasone-coated neural probes elicit attenuated inflammatory response and neuronal loss compared to uncoated neural probes, Brain Res 1148 (2007) 15–27. 10.1016/j.brainres.2007.02.024. 17376408 PMC1950487

[40] Kozai T.D.Y., Jaquins-Gerstl A.S., Vazquez A.L., Michael A.C., Cui X.T., Dexamethasone retrodialysis attenuates microglial response to implanted probes in vivo, Biomaterials 87 (2016) 157–169. 10.1016/j.biomaterials.2016.02.013. 26923363 PMC4866508

[41] Spataro L., Dilgen J., Retterer S., Spence A.J., Isaacson M., Turner J.N., Shain W., Dexamethasone treatment reduces astroglia responses to inserted neuroprosthetic devices in rat neocortex, Exp Neurol 194 (2005) 289–300. 10.1016/j.expneurol.2004.08.037. 16022859

[42] Potter-Baker K.A., Stewart W.G., Tomaszewski W.H., Wong C.T., Meador W.D., Ziats N.P., Capadona J.R., Implications of chronic daily anti-oxidant administration on the inflammatory response to intracortical microelectrodes, J Neural Eng 12 (2015). 10.1088/1741-2560/12/4/046002. PMC4510031 26015427

[43] Potter K.A., Jorfi M., Householder K.T., Foster E.J., Weder C., Capadona J.R., Curcumin-releasing mechanically adaptive intracortical implants improve the proximal neuronal density and blood-brain barrier stability, Acta Biomater 10 (2014) 2209–2222. 10.1016/j.actbio.2014.01.018. 24468582

[44] Mueller N.N., Kim Y., Ocoko M.Y.M., Dernelle P., Kale I., Patwa S., Hermoso A.C., Chirra D., Capadona J.R., Hess-Dunning A., Effects of micromachining on anti-oxidant elution from a mechanically-adaptive polymer, Journal of Micromechanics and Microengineering 34 (2024). 10.1088/1361-6439/ad27f7. PMC10996452 38586082

[45] Malkawi A.K., Alzoubi K.H., Jacob M., Matic G., Ali A., Al Faraj A., Almuhanna F., Dasouki M., Rahman A.M.A., Metabolomics based profiling of Dexamethasone side effects in rats, Front Pharmacol 9 (2018). 10.3389/fphar.2018.00046. PMC5820529 29503615

[46] Systemically delivered platelet-inspired nanoparticles to reduce inflammation surrounding intracortical microelectrodes_Longshun Li_09042024_clean, (n.d.).

[47] Ravikumar M., Modery C.L., Wong T.L., Sen Gupta A., Peptide-decorated liposomes promote arrest and aggregation of activated platelets under flow on vascular injury relevant protein surfaces in vitro, Biomacromolecules 13 (2012) 1495–1502. 10.1021/bm300192t. 22468641

[48] Modery-Pawlowski C.L., Tian L.L., Ravikumar M., Wong T.L., Sen Gupta A., In vitro and in vivo hemostatic capabilities of a functionally integrated platelet-mimetic liposomal nanoconstruct, Biomaterials 34 (2013) 3031–3041. 10.1016/j.biomaterials.2012.12.045. 23357371

[49] Hickman D.A., Pawlowski C.L., Shevitz A., Luc N.F., Kim A., Girish A., Marks J., Ganjoo S., Huang S., Niedoba E., Sekhon U.D.S., Sun M., Dyer M., Neal M.D., Kashyap V.S., Sen Gupta A., Intravenous synthetic platelet (SynthoPlate) nanoconstructs reduce bleeding and improve “golden hour” survival in a porcine model of traumatic arterial hemorrhage, Sci Rep 8 (2018). 10.1038/s41598-018-21384-z. PMC5814434 29449604

[50] Shoffstall A.J., Ecker M., Danda V., Joshi-Imre A., Stiller A., Yu M., Paiz J.E., Mancuso E., Bedell H.W., Voit W.E., Pancrazio J.J., Capadona J.R., Characterization of the neuroinflammatory response to thiol-ene shape memory polymer coated intracortical microelectrodes, Micromachines (Basel) 9 (2018). 10.3390/mi9100486. PMC6215215 30424419

[51] Hernandez-Reynoso A.G., Sturgill B.S., Hoeferlin G.F., Druschel L.N., Krebs O.K., Menendez D.M., Thai T.T.D., Smith T.J., Duncan J., Zhang J., Mittal G., Radhakrishna R., Desai M.S., Cogan S.F., Pancrazio J.J., Capadona J.R., The effect of a Mn(III)tetrakis(4-benzoic acid)porphyrin (MnTBAP) coating on the chronic recording performance of planar silicon intracortical microelectrode arrays, Biomaterials 303 (2023). 10.1016/j.biomaterials.2023.122351. PMC10842897 37931456

[52] Anderson R., Franch A., Castell M., Perez-Cano F.J., Bräuer R., Pohlers D., Gajda M., Siskos A.P., Katsila T., Tamvakopoulos C., Rauchhaus U., Panzner S., Kinne R.W., Liposomal encapsulation enhances and prolongs the anti-inflammatory effects of water-soluble dexamethasone phosphate in experimental adjuvant arthritis, Arthritis Res Ther 12 (2010). 10.1186/ar3089. PMC2945041 20642832

[53] Chen X.Y., Wang S.M., Li N., Hu Y., Zhang Y., Xu J.F., Li X., Ren J., Su B., Yuan W.Z., Teng X.R., Zhang R.X., hua Jiang D., Mulet X., Li H.P., Creation of Lung-Targeted Dexamethasone Immunoliposome and Its Therapeutic Effect on Bleomycin-Induced Lung Injury in Rats, PLoS One 8 (2013). 10.1371/journal.pone.0058275. PMC3597622 23516459

[54] Kim Y., Park E.J., Kim T.W., Na D.H., Recent progress in drug release testing methods of biopolymeric particulate system, Pharmaceutics 13 (2021). 10.3390/pharmaceutics13081313. PMC8399039 34452274

[55] Ereifej E.S., Li Y., Goss-Varley M., Kim Y., Meade S.M., Chen K., Rayyan J., Feng H., Dona K., McMahon J., Taylor D., Capadona J.R., Sun J., Investigating the association between motor function, neuroinflammation, and recording metrics in the performance of intracortical microelectrode implanted in motor cortex, Micromachines (Basel) 11 (2020). 10.3390/MI11090838. PMC7570280 32899336

[56] Sturgill B., Radhakrishna R., Thai T.T.D., Patnaik S.S., Capadona J.R., Pancrazio J.J., Characterization of Active Electrode Yield for Intracortical Arrays: Awake versus Anesthesia, Micromachines (Basel) 13 (2022). 10.3390/mi13030480. PMC8955818 35334770

[57] Usoro J.O., Dogra K., Abbott J.R., Radhakrishna R., Cogan S.F., Pancrazio J.J., Patnaik S.S., Influence of implantation depth on the performance of intracortical probe recording sites, Micromachines (Basel) 12 (2021). 10.3390/mi12101158. PMC8539313 34683209

[58] Stiller A.M., Usoro J., Frewin C.L., Danda V.R., Ecker M., Joshi-Imre A., Musselman K.C., Voit W., Modi R., Pancrazio J.J., Black B.J., Chronic intracortical recording and electrochemical stability of thiolene/acrylate shape memory polymer electrode arrays, Micromachines (Basel) 9 (2018). 10.3390/mi9100500. PMC6215160 30424433

[59] Moore A.K., Wehr M., A guide to in vivo single-unit recording from optogenetically identified cortical inhibitory interneurons, Journal of Visualized Experiments (2014). 10.3791/51757. PMC4353425 25407742

[60] Gage G.J., Kipke D.R., Shain W., Whole animal perfusion fixation for rodents., J Vis Exp (2012). 10.3791/3564. PMC3476408 22871843

[61] Ereifej E.S., Rial G.M., Hermann J.K., Smith C.S., Meade S.M., Rayyan J.M., Chen K., Feng H., Capadona J.R., Implantation of neural probes in the brain elicits oxidative stress, Front Bioeng Biotechnol 6 (2018). 10.3389/fbioe.2018.00009. PMC5816578 29487848

[62] Potter-Baker K.A., Ravikumar M., Burke A.A., Meador W.D., Householder K.T., Buck A.C., Sunil S., Stewart W.G., Anna J.P., Tomaszewski W.H., Capadona J.R., A comparison of neuroinflammation to implanted microelectrodes in rat and mouse models, Biomaterials 35 (2014) 5637–5646. 10.1016/j.biomaterials.2014.03.076. 24755527 PMC4071936

[63] Lindner S.C., Yu M., Capadona J.R., Shoffstall A.J., A graphical user interface to assess the neuroinflammatory response to intracortical microelectrodes HHS Public Access, J Neurosci Methods 317 (2019) 141–148. 10.1016/j.jneumeth. 30664915 PMC6914213

[64] Stringer C., Wang T., Michaelos M., Pachitariu M., Cellpose: a generalist algorithm for cellular segmentation, Nat Methods 18 (2021) 100–106. 10.1038/s41592-020-01018-x. 33318659

[65] Pachitariu M., Stringer C., Cellpose 2.0: how to train your own model, Nat Methods 19 (2022) 1634–1641. 10.1038/s41592-022-01663-4. 36344832 PMC9718665

[66] Serna-Rodríguez M.F., Bernal-Vega S., de la Barquera J.A.O.S., Camacho-Morales A., Pérez-Maya A.A., The role of damage associated molecular pattern molecules (DAMPs) and permeability of the blood-brain barrier in depression and neuroinflammation, J Neuroimmunol 371 (2022). 10.1016/j.jneuroim.2022.577951. 35994946

[67] Roh J.S., Sohn D.H., Damage-associated molecular patterns in inflammatory diseases, Immune Netw 18 (2018). 10.4110/in.2018.18.e27. PMC6117512 30181915

[68] Song S., Regan B., Ereifej E.S., Chan E.R., Capadona J.R., Neuroinflammatory Gene Expression Analysis Reveals Pathways of Interest as Potential Targets to Improve the Recording Performance of Intracortical Microelectrodes, Cells 11 (2022). 10.3390/cells11152348. PMC9367362 35954192

[69] Kumar H., Kawai T., Akira S., Pathogen recognition by the innate immune system, Int Rev Immunol 30 (2011) 16–34. 10.3109/08830185.2010.529976. 21235323

[70] Collinger J.L., Wodlinger B., Downey J.E., Wang W., Tyler-Kabara E.C., Weber D.J., McMorland A.J.C., Velliste M., Boninger M.L., Schwartz A.B., High-performance neuroprosthetic control by an individual with tetraplegia, The Lancet 381 (2013) 557–564. 10.1016/S0140-6736(12)61816-9. PMC3641862 23253623

[71] King C.E., Wang P.T., Chui L.A., Do A.H., Nenadic Z., Operation of a brain-computer interface walking simulator for individuals with spinal cord injury, 2013. http://www.jneuroengrehab.com/content/10/1/77http://www.jneuroengrehab.com/content/10/1/77. 10.1186/1743-0003-10-77 PMC3723437 23866985

[72] Rupp R., Challenges in clinical applications of brain computer interfaces in individuals with spinal cord injury, Front Neuroeng 7 (2014). 10.3389/fneng.2014.00038. PMC4174119 25309420

[73] Alam M., Rodrigues W., Pham B.N., Thakor N. V., Brain-machine interface facilitated neurorehabilitation via spinal stimulation after spinal cord injury: Recent progress and future perspectives, Brain Res 1646 (2016) 25–33. 10.1016/j.brainres.2016.05.039. 27216571

[74] Collinger J.L., Boninger M.L., Bruns T.M., Curley K., Wang W., Weber D.J., Bruns M., Curley K., Wang W., Weber D.J., Functional Priorities, Assistive Technology, and Brain-Computer Interfaces after Spinal Cord Injury, 2013. 10.1682/jrrd.2011.11.0213 PMC3684986 23760996

[75] Kozai T.D.Y., Jaquins-Gerstl A.S., Vazquez A.L., Michael A.C., Cui X.T., Brain tissue responses to neural implants impact signal sensitivity and intervention strategies, ACS Chem Neurosci 6 (2015) 48–67. 10.1021/cn500256e. 25546652 PMC4304489

[76] Kozai T.D.Y., Gugel Z., Li X., Gilgunn P.J., Khilwani R., Ozdoganlar O.B., Fedder G.K., Weber D.J., Cui X.T., Chronic tissue response to carboxymethyl cellulose based dissolvable insertion needle for ultra-small neural probes, Biomaterials 35 (2014) 9255–9268. 10.1016/j.biomaterials.2014.07.039. 25128375

[77] Szarowski D.H., Andersen M.D., Retterer S., Spence A.J., Isaacson M., Craighead H.G., Turner J.N., Shain W., Brain responses to micro-machined silicon devices, Brain Res 983 (2003) 23–35. 10.1016/S0006-8993(03)03023-3. 12914963

[78] McConnell G.C., Rees H.D., Levey A.I., Gutekunst C.A., Gross R.E., Bellamkonda R. V., Implanted neural electrodes cause chronic, local inflammation that is correlated with local neurodegeneration, J Neural Eng 6 (2009). 10.1088/1741-2560/6/5/056003. 19700815

[79] Stence N., Waite M., Dailey M.E., Dynamics of microglial activation: A confocal time-lapse analysis in hippocampal slices, Glia 33 (2001) 256–266. 10.1002/1098-1136(200103)33:3&lt;256::AID-GLIA1024&gt;3.0.CO;2-J. 11241743

[80] Biran R., Martin D.C., Tresco P.A., Neuronal cell loss accompanies the brain tissue response to chronically implanted silicon microelectrode arrays, Exp Neurol 195 (2005) 115–126. 10.1016/j.expneurol.2005.04.020. 16045910

[81] Chang N.P., DaPrano E.M., Lindman M., Estevez I., Chou T.W., Evans W.R., Nissenbaum M., McCourt M., Alzate D., Atkins C., Kusnecov A.W., Huda R., Daniels B.P., Neuronal DAMPs exacerbate neurodegeneration via astrocytic RIPK3 signaling, JCI Insight 9 (2024). 10.1172/jci.insight.177002. PMC11382884 38713518

[82] Zhang X., Khan S., Wei R., Zhang Y., Liu Y., Wee Yong V., Xue M., Application of nanomaterials in the treatment of intracerebral hemorrhage, J Tissue Eng 14 (2023). 10.1177/20417314231157004. PMC10074624 37032735

[83] Chen Y., Chen X., Zhou Q., Different effects of a perioperative single dose of dexamethasone on wound healing in mice with or without sepsis, Front Surg 10 (2023). 10.3389/fsurg.2023.927168. PMC10126451 37114154

[84] Barnes P.J., Karin M., Nuclear Factor-κB — A Pivotal Transcription Factor in Chronic Inflammatory Diseases, New England Journal of Medicine 336 (1997) 1066–1071. 10.1056/nejm199704103361506. 9091804

[85] Castro-Caldas M., Mendes A.F., Carvalho A.P., Duarte C.B., Lopes M.C., Dexamethasone prevents interleukin-1β-induced nuclear factor-κB activation by upregulating IκB-α synthesis, in lymphoblastic cells, Mediators Inflamm 12 (2003) 37–46. 10.1080/0962935031000096953. 12745547 PMC1781587

[86] Crinelli R., Antonelli A., Bianchi M., Gentilini L., Scaramucci S., Magnani M., Selective inhibition of NF-kB activation and TNF-α production in macrophages by red blood cell-mediated delivery of dexamethasone, Blood Cells Mol Dis 26 (2000) 211–222. 10.1006/bcmd.2000.0298. 10950941

[87] Song S., Druschel L.N., Chan E.R., Capadona J.R., Differential expression of genes involved in the chronic response to intracortical microelectrodes, Acta Biomater 169 (2023) 348–362. 10.1016/j.actbio.2023.07.038. 37507031 PMC10528922

[88] Chini E.N., Sen S., Eduardo, Chini N., And, Brown M.J., Complications After Unintentional Intra-arterial Injection of Drugs: Risks, Outcomes, and Management Strategies, 2005. www.mayoclinicproceedings.com783. 10.1016/S0025-6196(11)61533-4 15945530

